# Dietary fat intake and risk of Parkinson disease: results from the Swedish National March Cohort

**DOI:** 10.1007/s10654-022-00863-8

**Published:** 2022-04-13

**Authors:** Essi Hantikainen, Elin Roos, Rino Bellocco, Alessia D’Antonio, Alessandra Grotta, Hans-Olov Adami, Weimin Ye, Ylva Trolle Lagerros, Stephanie Bonn

**Affiliations:** 1 Department of Statistics and Quantitative Methods, University of Milano-Bicocca, 20126 Milan, Italy; 2 Institute for Biomedicine, (Affiliated to the University of Lübeck), Eurac Research, 39100 Bolzano, Italy; 3 Department of Global Public Health, Karolinska Institutet, 171 77 Stockholm, Sweden; 4 Department of Medical Epidemiology and Biostatistics, Karolinska Institutet, 171 77 Stockholm, Sweden; 5 Department of Public Health Sciences, Stockholm University, 106 91 Stockholm, Sweden; 6 Clinical Effectiveness Group, Institute of Health and Society, University of Oslo, 0318 Oslo, Norway; 7 Division of Clinical Epidemiology, Department of Medicine (Solna), Karolinska Institutet, 171 76 Stockholm, Sweden; 8 Center for Obesity, Academic Specialist Center, Stockholm Health Services, 113 65 Stockholm, Sweden

**Keywords:** Diet, Energy intake, Epidemiology, Fatty acids, Parkinson disease

## Abstract

**Background:**

Following progressive aging of the population worldwide, the prevalence of Parkinson disease is expected to increase in the next decades. Primary prevention of the disease is hampered by limited knowledge of preventable causes. Recent evidence regarding diet and Parkinson disease is inconsistent and suggests that dietary habits such as fat intake may have a role in the etiology.

**Objective:**

To investigate the association between intake of total and specific types of fat with the incidence of Parkinson disease.

**Methods:**

Participants from the Swedish National March Cohort were prospectively followed-up from 1997 to 2016. Dietary intake was assessed at baseline using a validated food frequency questionnaire. Food items intake was used to estimate fat intake, i.e. the exposure variable, using the Swedish Food Composition Database. Total, saturated, monounsaturated and polyunsaturated fat intake were categorized into quartiles. Parkinson disease incidence was ascertained through linkages to Swedish population-based registers. Cox proportional hazards regression models were used to estimate hazard ratios (HR) with 95% confidence intervals (CI) of the association between fat intake from total or specific types of fats and the incidence of Parkinson disease. The lowest intake category was used as reference. Isocaloric substitution models were also fitted to investigate substitution effects by replacing energy from fat intake with other macronutrients or specific types of fat.

**Results:**

41,597 participants were followed up for an average of 17.6 years. Among them, 465 developed Parkinson disease. After adjusting for potential confounders, the highest quartile of saturated fat intake was associated with a 41% increased risk of Parkinson disease compared to the lowest quartile (HR Q4 vs. Q1: 1.41; 95% CI: 1.04–1.90; *p* for trend: 0.03). Total, monounsaturated or polyunsaturated fat intake were not significantly associated with Parkinson disease. The isocaloric substitution models did not show any effect.

**Conclusions:**

We found that a higher consumption of large amounts of saturated fat might be associated with an increased risk of Parkinson disease. A diet low in saturated fat might be beneficial for disease prevention.

**Supplementary Information:**

The online version contains supplementary material available at 10.1007/s10654-022-00863-8.

## Introduction

As the second most common neurodegenerative disorder, Parkinson disease affects approximately 1% of individuals older than 60 years [1]. With a growing aging population, an increase in the number of cases is expected [2]. The clinical characteristics including resting tremor, rigidity and bradykinesia seriously affects patients’ quality of life. Determining modifiable lifestyle factors causally associated with Parkinson disease is required for primary prevention.

The etiology of Parkinson disease is not well understood. However, epidemiological studies have shown that lifestyle and environmental factors are important for both disease pathogenesis and progression [3–6]. Some factors have indeed consistently been associated with Parkinson disease. A positive association has been noted for smoking, caffeine intake, and physical activity, and a negative association for dairy intake [6, 7]. To what extent dietary choices have an influence on the development of Parkinson disease has been discussed. A Mediterranean diet seems to be inversely associated with both prodromal features of Parkinson disease [8] and with the disease itself [9]. There are several theories regarding the role of fat intake in Parkinson disease. Oxidative stress and neuro-inflammation may be involved in the pathogenesis of the disease [10–12]. Polyunsaturated fatty acids have shown both anti-inflammatory, anti-apoptotic, anti-oxidant and neuroprotective properties, and monounsaturated fatty acids seem able to reduce oxidative stress [5]. Animal model studies of Parkinson disease suggest that elevated dietary levels of omega-3, a type of polyunsaturated fatty acids, protect against neuroinflammation and dopaminergic damage [13, 14].

Findings on the association between dietary fat intake and Parkinson disease risk are inconsistent [15–23] with no clear evidence of an association between intake of total and specific types of fat and Parkinson disease risk. The latest published meta-analysis showed an association with high total energy intake and Parkinson disease incidence, but the statistical significance was too weak to ensure an association with total and specific types of fat intakes [24]. The authors recommended future research to focus on specific types of dietary fat. We therefore aimed to further investigate the topic by using prospective data from a large Swedish cohort, including information on specific types of dietary fat.

## Materials and methods

### Study population

The Swedish National March Cohort includes 43,865 Swedish participants who, at a cancer research fund-raising event conducted in 1997, were asked to complete a 36-page questionnaire including lifestyle factors, medical history and a detailed assessment of dietary habits. The questionnaire has previously been described in detail [25]. Participants were encouraged to fill in the questionnaire at home. Self-reported information on additional lifestyle factors such as physical activity, tobacco use and alcohol intake, as well as height, weight, diabetes and educational level, was also assessed. All participants provided their individually unique national registration number, which allowed for identification of health status through record linkages to Swedish population-based registers including the National Patient Register and the Cause of Death Register. Parkinson disease diagnosis is validated in the registers, showing high sensitivity and accuracy when compared to clinical data [26].

Participants who had wrong national registration numbers (n = 11), who were below 18 years of age at baseline (n = 1741), who already had been diagnosed with Parkinson disease (n = 10), who had emigrated (n = 41) or died (n = 8) before the start of follow-up were excluded from the analysis. We further excluded 459 participants for whom values of total caloric intake were deemed unrealistic, i.e. were larger or smaller than ± 3 standard deviations for the sex-specific mean value of log_e_ transformed energy. The final cohort included 41,597 participants.

### Assessment of dietary intake

Dietary intake at baseline was assessed with an 85-item validated semi-quantitative, self-administered food frequency questionnaire (FFQ) [27]. Participants were asked to report how often, on average, they consumed each food item and beverage included in the FFQ. Depending on the type of consumed item, answers consisted of eight response options ranging from 0 to 7 times or more per day, or categorized as: 0, 1–3 times/month; 1–2, 3–4, 5–6 times/week or 1, 2, 3 times/day. Intake of total energy (kcal/day), total fat (g/day), as well as saturated, monounsaturated and polyunsaturated fat (g/day) was calculated by linking the FFQ to the Swedish Food Composition Database held by the National Food Agency of Sweden [28]. Intake of nutrients was energy-adjusted using the nutrient residual method [29]. In brief, the method aims at adjusting for confounding and removing extraneous variation by energy intake, since the intake of macronutrients is highly correlated with total energy intake. Fat intake (total, saturated, monounsaturated, and polyunsaturated) was categorized into quartiles based on the individual distributions among women and men separately.

### Outcome

Participants in the cohort were followed from baseline until diagnosis of Parkinson disease, emigration, death or end of follow-up on December 31st, 2016, whichever occurred first. Incident cases of Parkinson disease were identified through linkage to the National Patient Register. The date of diagnosis was defined as first ever outpatient contact or hospital discharge with a diagnosis of Parkinson disease registered in the Swedish Patient Register. The Swedish Revisions of the International Classification of Diseases (ICD) codes for Parkinson disease were used: 350 (ICD-7, 1964–68), 342 (ICD-8, 1969–86), 332A (ICD-9, 1987–96), and G20 (ICD-10, 1997–0).

### Statistical analysis

Energy-adjusted intake of macronutrients and that of polyunsaturated-, monounsaturated- and saturated fats was calculated by implementing the nutrient residual method to control for either possible confounding or extraneous variability [29]. Descriptive statistics of the study cohort were presented by quartiles of total fat intake. Age adjusted incidence rates were calculated based on the 5-year age categories distribution of follow-up person-years of the entire population.

Cox proportional hazards regression models, with attained age as the underlying time scale, were used to obtain hazard ratios (HR) with 95% confidence intervals (CI) comparing quartile categories of fat intake with the lowest quartile as the reference. Crude models were age and sex-adjusted. Based on subject matter knowledge, we additionally adjusted the multivariable models for the following potential confounders: Daily intake of coffee (categories of 0, 1–2, 3–4, and ≥ 5 cups/day) and intake of Vitamin E (mg/day) assessed using the FFQ, Body mass index (BMI, kg/m^2^) calculated using self-reported weight and height at baseline, level of education (≤ 13 or > 13 years of education), smoking status (never, former or current) and physical activity, including household and commuting activity (categories of ≤ 2, 3–4, 5–6, > 6 h/week). Linear trends were investigated by estimating the median value of each quartile of dietary fat intake and specific types of fat, respectively, and by implementing these as continuous variables in each model.

The validity of the proportional hazards assumption was assessed using scaled Schoenfeld residuals [30]. We further investigated potential effect modification of the relationship between fat intake, specific types of fat and Parkinson disease by sex on the multiplicative scale. To do so we fitted models including the cross-product interaction terms between sex (males, females) and quartiles of each exposure, and used the likelihood ratio test to compare nested models. The dose–response relationship was analyzed by conducting restricted cubic splines with four knots at the 5, 35, 65 and 95th percentile of the distribution of each exposure [31].

We further investigated the effect of replacing total fat intake by other macronutrients and specific types of fat based on data from our cohort. To do so, intake of total and specific types of the macronutrients; fat, carbohydrates, protein, and alcohol, were calculated as percentages of the total energy intake. We then fitted isocaloric models [32] to estimate the effect of substituting 10% of energy coming from fat with the same amount of energy derived from carbohydrates, proteins or alcohol; and the effect of substituting 10% of energy from one type of fat (saturated, monounsaturated, or polyunsaturated) with an isocaloric amount derived from one of the other types of fat.

Food substitution modelling using dietary data assessed at a single time point has been described in detail by Ibsen et al. [33]. More specifically, as more than three types of macronutrients were considered for substitution analysis we used the leave-one-out model described by Song and Giovannucci [34] to calculate the associations with Parkinson disease risk for substituting one macronutrient for another. In brief, to estimate the effect on outcome Y of substituting macronutrients A and B for C while keeping total A, B and C consumption constant, C was left out of a multivariable model including both the total consumption of A, B and C, and the individual consumptions of A and B (Y = A + B + (A + B + C)).

Replacement of ten percent of energy was chosen to reflect a feasible change related to total energy intake. Replacing 10% of energy from macronutrients would yield an amount that would be feasible to substitute in terms of actual intake. For example, the substituted nutrient, e.g. total fat, was not included in the model, while the remaining sources of energy, i.e. carbohydrates, proteins and alcohol, and total energy intake, were added as confounders. The coefficients for carbohydrate, protein and alcohol in the example therefore represent the difference in effect of substituting an equal amount of energy from fat by carbohydrate, protein and alcohol, when keeping the total energy intake constant.

Several sensitivity analyses were conducted to assess the robustness of our findings. First, we repeated the analyses by also including cases from the Cause of Death Register. In addition, we excluded cases occurring during the first three, or five years of follow-up to investigate a possible effect due to reverse causation. Finally, we imputed missing values using multiple imputation based on chained equations [35].

P-values lower than 0.05 were considered statistically significant. All statistical analyses were performed using SAS 9.4 and Stata, Version 15.1 (StataCorp LP, College Station, Texas).

## Results

Characteristics of study participants by quartiles of total fat intake are shown in Table 1. Among the 41,597 study participants, 26,754 (64.3%) were women and 14,843 (35.7%) were men. Average follow-up time was 17.6 years, with an overall 732,966 person-years for the cohort. During follow-up, 465 participants were diagnosed with Parkinson disease. At baseline, the mean age was 51.6 (SD 16.0) years. The median (IQR) percentages of energy from fat intake in the quartiles were: 20.3 (2.3), 24.2 (0.8), 26.7 (0.8) and 30.5 (2.1) from the lowest to the highest quartile, respectively. The mean total energy intake reported in the FFQ was 2,038 (SD 534) kcal/day. On average, most of the energy came from carbohydrates (56.6%, SD 4.8). Fat, proteins and alcohol contributed with 25.4% (SD 4.1), 16.0% (SD 1.9) and 3.7% (SD 5.7) to the total energy intake, respectively. Participants with higher total fat intake tended to be obese and smokers, and to have fewer years of education. Participants with lower fat intake were more physically active and consumed less coffee.

**Table 1:** Baseline characteristics of study participants in the Swedish National March Cohort by quartiles of energy-adjusted total fat intake

	Total	Quartiles of Total Fat Intake			
		Q1	Q2	Q3	Q4
*Males range of fat intake, g/day, Mean (sd), by quartile*	55.54 (8.61)	14.01–50.08	50.09–55.49	50.50–61.00	60.01–127.37
*Females range of fat intake, g/day, Mean (sd), by quartile*	55.85 (9.03)	16.01–50.13	50.14–56.00	56.01–61.83	61.84–111.32
N	41 597	10 400	10 399	10 400	10 398
Males, N (%)	14 843 (35.7)	3711 (35.7)	3711 (35.7)	3711 (35.7)	3710 (35.7)
Age, years, Mean (sd)	51.6 (16.0)	52.1 (16.2)	51.9 (15.9)	51.1 (16.0)	51.0 (15.9)
Body Mass Index (kg/m^2^), N (%)					
Underweight-Normal weight (≤ 24.9)	23 817 (59.9)	6047 (60.8)	5962 (59.9)	5958 (59.9)	5850 (59.2)
Overweight (25.0–29.9)	13 037 (32.8)	3175 (31.9)	3283 (33.0)	3301 (33.2)	3278 (33.1)
Obese (≥ 30.0)	2893 (7.3)	725 (7.3)	714 (7.2)	692 (7.0)	762 (7.7)
Smoking status, N (%)					
Never	24 625 (64.5)	6024 (63.7)	6202 (64.8)	6282 (65.5)	6117 (64.2)
Past	10 569 (27.7)	2776 (29.3)	2681 (28.0)	2564 (26.7)	2548 (26.7)
Current	2966 (7.8)	665 (7.0)	686 (7.2)	752 (7.8)	863 (9.1)
Coffee Intake, N (%)					
0 cups/day	4958 (12.1)	1452 (14.2)	1174 (11.5)	1176 (11.5)	1156 (11.3)
1–2 cups/day	12 248 (30.0)	3183 (31.2)	3084 (30.1)	3074 (30.1)	2907 (28.5)
3–4 cups/day	16 653 (40.7)	3906 (38.3)	4329 (42.3)	4242 (41.5)	4176 (40.9)
≥ 5 cups/day	7029 (17.2)	1668 (16.3)	1653 (16.1)	1733 (17.0)	1975 (19.3)
E-Vitamin Intake, mg/day, Mean (sd)	5.23 (1.2)	5.36 (1.4)	5.19 (1.1)	5.15 (1.0)	5.21 (1.0)
Physical Activity					
≤ 2 h/week	6397 (15.5)	1496 (14.5)	1489 (14.4)	1624 (15.7)	1788 (17.3)
3–4 h/week	12 274 (29.7)	2986 (28.9)	3097 (29.9)	3090 (29.9)	3101 (30.0)
5–6 h/week	9156 (22.1)	2309 (22.3)	2350 (22.7)	2288 (22.1)	2209 (21.4)
> 6 h/week	13 565 (32.8)	3557 (34.4)	3415 (33.0)	3343 (32.3)	3250 (31.4)
Education, years N (%)					
≤ 13 years	29 214 (71.6)	6954 (68.1)	7224(70.9)	7382 (72.3)	7654 (75.1)
> 13 years	11 590 (28.4)	3251 (31.9)	2972 (29.2)	2827 (27.7)	2540 (24.9)
Energy Intake, kcal/day, Mean (sd) % of Energy Intake, Mean (sd), from:	2038 (534)	2012 (530)	2068 (522)	2064 (530)	2008 (552)
Fats	25.4 (4.1)	20.3 (2.3)	24.2 (0.8)	26.7 (0.8)	30.5 (2.1)
Proteins	16.0 (1.9)	15.7 (2.2)	15.9 (1.9)	16.1 (1.7)	16.4 (1.8)
Carbohydrates	56.6 (4.8)	61.9 (3.6)	57.9 (2.4)	55.3 (2.3)	51.4 (3.1)
Alcohol	3.7 (5.7)	3.8 (7.6)	3.6 (5.1)	3.6 (5.0)	3.6 (4.9)
% of Energy Intake, Mean (sd), from:					
Saturated Fats	11.4 (2.4)	8.7 (1.4)	10.7 (1.0)	12.1 (1.1)	14.1 (1.6)
Monounsaturated Fats	8.5 (1.5)	6.7 (0.9)	8.1 (0.6)	8.9 (0.6)	10.2 (1.0)
Polyunsaturated Fats	3.3 (0.6)	3.0 (0.5)	3.3 (0.5)	3.4 (0.5)	3.6 (0.6)

**Fig. 1:**
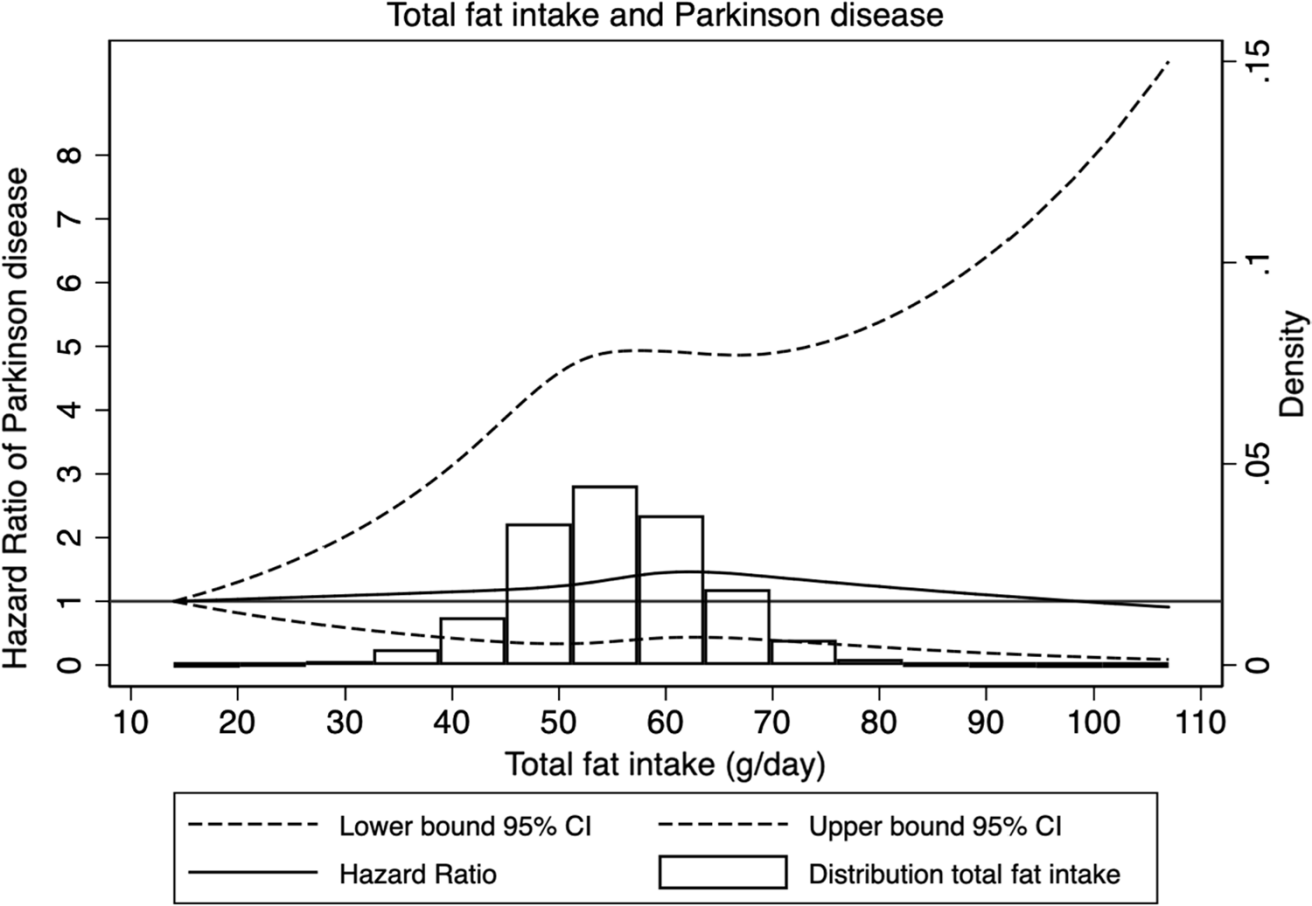
Multivariable-adjusted restricted cubic spline curve for the association between dietary intake from total fat, measured in g/day, and the risk of Parkinson disease. Adjustments were made for age (underlying time scale), sex, intake of coffee (categories of 0, 1–2, 3–4, and ≥ 5 cups/day), dietary intake of Vitamin E (mg/day), Body mass index (BMI, kg/m2), education (≤ 13 or > 13 years), smoking status (never, former or current) and physical activity, including household and commuting activity (categories of ≤ 2, 3–4, 5–6, > 6 h/week)

**Fig. 2:**
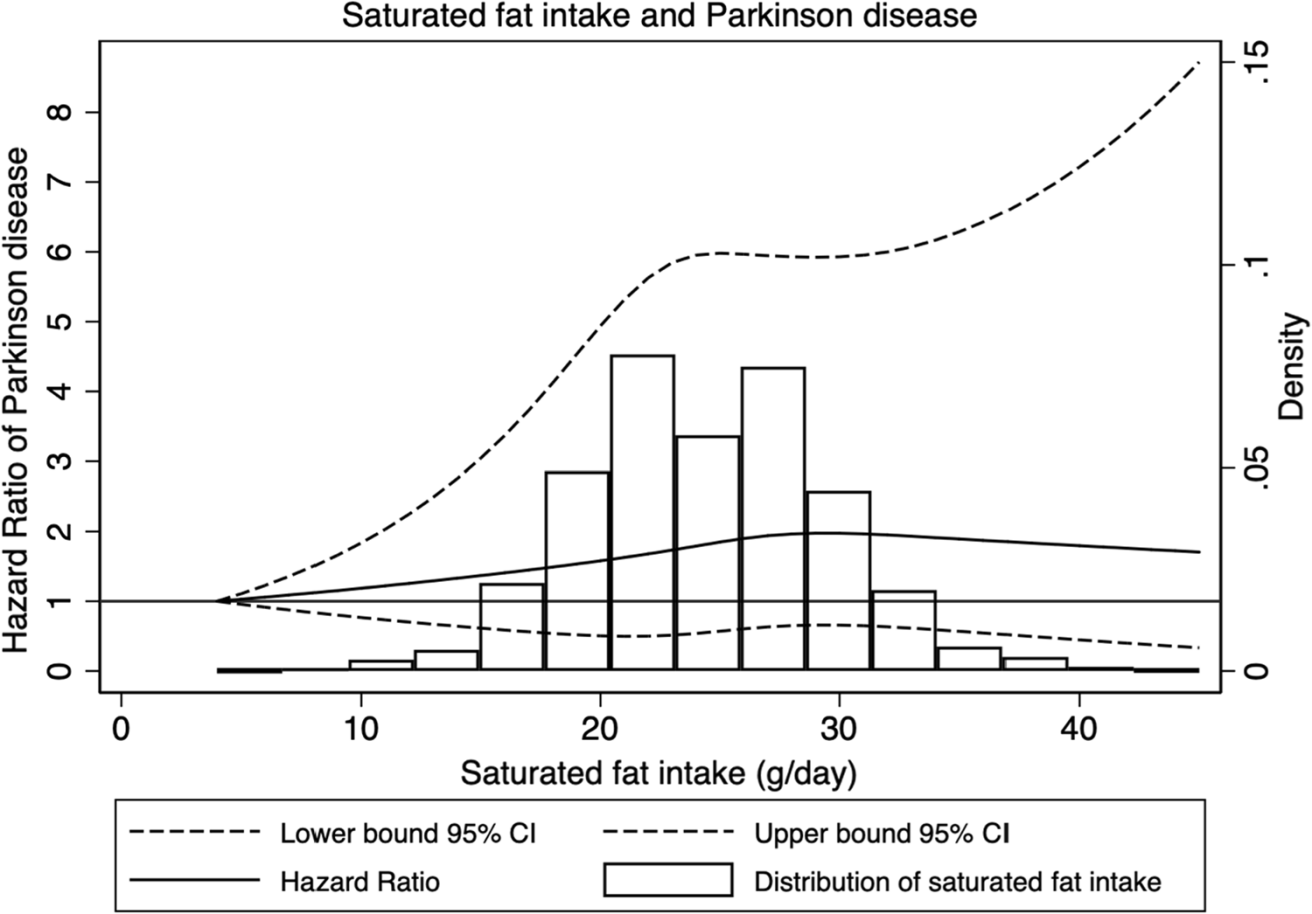
Multivariable-adjusted restricted cubic spline curve for the association between dietary intake from saturated fat, measured in g/day, and the risk of Parkinson disease. Adjustments were made for age (underlying time scale), sex, intake of coffee (categories of 0, 1–2, 3–4, and ≥ 5 cups/day), dietary intake of Vitamin E (mg/day), Body mass index (BMI, kg/m2), education (≤ 13 or > 13 years), smoking status (never, former or current) and physical activity, including household and commuting activity (categories of ≤ 2, 3–4, 5–6, > 6 h/week)

Results from the analysis of total fat intake and intake of saturated fat are shown in Table 2. In the age and sex-adjusted model an association was found with total fat intake (HR Q4 vs. Q1: 1.29; 95% CI: 1.00–1.68; *p* for trend: 0.05) and intake of saturated fat (HR Q4 vs. Q1: 1.41; 95% CI: 1.08–1.85; *p* for trend: 0.01) and the risk of Parkinson disease. After adjusting for potential confounders, this association was not significant anymore for total fat intake (HR: 1.29; 95% CI: 0.96–1.74; *p* for trend: 0.08). The multivariable adjusted model for categories of saturated fat intake showed a significant increased risk of Parkinson disease among participants in the higher quartiles compared to the lowest, with a HR of 1.38 (95% CI: 1.02–1.86) in Q2 vs. Q1, a HR of 1.45 (95% CI: 1.07–1.95) in Q3 vs. Q1, and a HR of 1.41 (95% CI: 1.04–1.90) in Q4 vs. Q1, and a significant trend (*p* for trend: 0.03). The multivariable adjusted model for continuous saturated fat intake showed an increased risk of Parkinson disease per each 1 SD increment (HR 1.10; 95% CI: 1.00–1.22). In addition, spline analyses did not reveal any deviation from a linear association (*p* for non-linearity: 0.19) (Figs. 1, 2, and supplementary figures S1-S2), indicating that the dose–response relationship might indeed be linear. No statistically significant associations were seen for the intake of monounsaturated (*p* for trend: 0.85), or polyunsaturated fats (*p* for trend: 0.66) and the risk of Parkinson disease (Table 2). When investigating potential effect modification by sex, we did not detect any evidence for an interaction between sex and intake of saturated fat (*p*-value for LR test: 0.48).

**Table 2:** Multivariable-adjusted hazard ratios with 95% confidence intervals for Parkinson disease risk by total fat intake and saturated fat intake, N = 41,597, Swedish National March Cohort, 1997–2016

	n	Person years	Incidence rates (per 100,000)^a^	Age and sex adjusted		Multivariable adjusted	
				HR	95% CI	HR	95% CI
Total Fat Intake, *g/day*							
Continuous, *per each 1 SD increment*				1.06	0.97–1.16	1.07	0.96–1.19
Q1	105	182,415	76.55	1	(Reference)	1	(Reference)
Q2	119	183,481	82.92	1.17	0.90–1.52	1.24	0.93–1.66
Q3	118	183,707	103.73	1.22	0.94–1.59	1.30	0.97–1.74
Q4	123	183,364	99.60	1.29	1.00–1.68	1.29	0.96–1.74
* p* for trend					0.05		0.08
Saturated Fat Intake, *g/day*							
Continuous, *per each 1 SD increment*				1.11	1.02–1.22	1.10	1.00–1.22
Q1	94	183,763	63.78	1	(Reference)	1	(Reference)
Q2	120	183,428	83.14	1.30	1.00–1.71	1.38	1.02–1.86
Q3	127	183,431	95.50	1.45	1.11–1.90	1.45	1.07–1.95
Q4	124	182,344	112.79	1.41	1.08–1.85	1.41	1.04–1.90
* p* for trend					0.01		0.03
Polyunsaturated Fat Intake, *g/day*							
Continuous, *per each 1 SD increment*				0.92	0.84–1.01	0.97	0.87–1.08
Q1	118	181,764	114.90	1	(Reference)	1	(Reference)
Q2	119	183,281	85.88	0.99	0.77–1.28	1.13	0.85–1.50
Q3	116	183,754	80.46	0.94	0.73–1.22	1.08	0.80–1.44
Q4	112	184,167	68.44	0.89	0.69–1.16	1.05	0.78–1.42
*vp* for trend					0.35		0.85
Monounsaturated Fat Intake, *g/day*							
Continuous, *per each 1 SD increment*				1.03	0.94–1.13	1.03	0.93–1.14
Q1	126	180,892	87.28	1	(Reference)	1	(Reference)
Q2	111	183,000	76.29	0.95	0.73–1.22	0.95	0.72–1.26
Q3	115	184,162	110.32	1.08	0.84–1.39	1.03	0.77–1.36
Q4	113	184,911	88.89	1.10	0.85–1.42	1.05	0.79–1.40
* p* for trend					0.35		0.66

*N * Number of Parkinson disease cases; *HR* Hazard Ratio; *CI* Confidence Interval

^a^Age adjusted incidence rates, based on 5-year age categories distribution of follow-up person-years of the entire population

Analyses performed with Cox Proportional Hazards regression model, with attained age as timescale, adjusted for sex (male/female), coffee intake (0, 1–2, 3–4, ≥ 5 cups/day), physical activity (≤ 2, 3–4, 5–6, > 6 h/week), smoking (never, former, current), BMI (kg/m^2^), education (≤ 13 years/ > 13 years), intake of vitamin-E (mg/day) and total energy intake (kcal)

Results from the isocaloric substitution models are shown in Table 3. We did not detect any significant effect by replacing 10% of fat intake with an equivalent amount of energy from carbohydrates, protein or alcohol. Similarly, no significant effect was found by replacing 10% of the energy intake from specific types of fat with other types of fat. Although not significant, replacing 10% of the total energy intake from polyunsaturated fat or monounsaturated fat, with saturated fat, was associated with a higher risk of Parkinson Disease, with the HRs being 1.76 (95% CI: 0.65–4.76) and 1.81 (95% CI: 0.56–5.83), respectively.

**Table 3:** Multivariable-adjusted hazard ratios with 95% confidence intervals for Parkinson disease risk from isocaloric substitution models, when replacing 10% of the total energy from total fat with the same amount of energy from other macronutrients or when replacing 10% of the total energy from individual fats with the same amount of energy from other types of fats

Substitution of:	HR	95% CI
10% of the total energy from fat with:		
Proteins	0.93	0.50–1.75
Carbohydrates	0.85	0.66–1.11
Alcohol	0.87	0.67–1.13
Substitution of:		
10% of the total energy from saturated fat with:		
Monounsaturated fat	0.37	0.06–2.32
Polyunsaturated fat	0.44	0.05–4.15
10% of the total energy from monounsaturated fat with:		
Saturated fat	1.81	0.56–5.83
Polyunsaturated fat	0.61	0.04–8.44
10% of the total energy from polyunsaturated fat with:		
Saturated fat	1.76	0.65–4.76
Monounsaturated fat	0.0	0.07–2.41

*HR*  Hazard Ratio; *CI*  Confidence Interval

Analyses performed with Cox Proportional Hazards regression model, with attained age as timescale, adjusting for sex (male/female), coffee intake (0, 1–2, 3–4, ≥ 5 cups/day), physical activity (≤ 2, 3–4, 5–6, > 6 h/week), smoking (never, former, current), intake of vitamin-E (mg/day) and total energy intake (kcal)

Conducting sensitivity analyses by additionally including cases from the Cause of Death register (additional cases n = 16), or after excluding cases occurring during the first three (n = 10) or five (n = 61) years of follow-up did not essentially affect our findings (results not shown). However, when excluding cases occurring during the first five years of follow-up, the multivariable adjusted HR´s remained significant only for saturated fat intake Q4 versus Q1: HR 1.39 (95% CI 1.01–1.92) with a non-significant *p for trend (p* for trend = 0.07). Percentages of missing values were equal to 8.3% for smoking status, 4.5% for BMI, 1.9% for education, 1.7% for coffee consumption, 1.0% for level of education, 0.5% for physical activity and 0.2% for alcohol consumption. After imputing missing values estimates remained similar (results not shown).

## Discussion

The present prospective cohort study including 41,597 participants investigated the association between dietary fat intake and Parkinson disease incidence. A high intake of saturated fat was associated with an increased Parkinson disease risk. No such association was found with intake of total fat, monounsaturated- or polyunsaturated fats.

Our results on saturated fats support previous findings from both cohort [15] and case control studies [16, 36]. Gao et al. investigated dietary patterns rather than specific single food items in a large prospective cohort study of 131,368 participants. Confounding adjustment and follow-up periods in their study were similar to our study. They found a lower risk for Parkinson disease with a prudent diet, which is a modification of the Mediterranean diet, with a low intake of saturated fats (also assessed by a FFQ, but linked to an American Food Composition Database), moderate intake of alcohol, and high intake of vegetables, legumes, fruits, whole grains, fish, nuts and poultry. Moreover, in the Nurses’ Health Study and the Health Professionals Follow-up Study a 5% isocaloric replacement of polyunsaturated fat with saturated fat was associated with an increased risk of Parkinson disease in men (RR: 1.83, 95% CI: 1.10–3.03) [21], even though the association was attenuated when excluding the first 6 years of follow-up. However, no statistically significant association was found in our study when replacing 10% of saturated fat with other fatty acids, although the HR´s were pointing in the same direction for polyunsaturated and monounsaturated fats. When replacing 10% of the total energy from fat with any other macronutrient, the effect sizes were, after all, pointing in the same direction, towards an inverse association with Parkinson disease, even though non-significant. The rather large confidence intervals in our substitution model may be driven by the distribution of the exposure variables, sample size or number of cases. We may not have enough power in the substitution model to look at specific types of fat. In addition, the food frequency questionnaire used to assess diet was not designed to measure fat intake specifically and random error cannot be ruled out. This could also have diluted the results.

The difference in effect sizes between our study and the Nurses’ Health Study and the Health Professionals Follow-up Study, both in main analyses and isocaloric substitution models, are likely due to several study design aspects. First, different FFQ's were used to assess exposure, and the nutrient composition of food was mainly estimated using the Harvard University Food Composition Database derived from the US Department of Agriculture, as compared to our Swedish Food Composition Database held by the National Food Agency of Sweden. Second, Parkinson disease was defined in slightly different ways. Third, the effect size on the same quantity of fat on the incidence of Parkinson disease may differ between participants in the Nurses´ Health Study and the Health Professionals Follow-up Study and our study due to differences in study populations, with potential different fat absorptions due to e.g. ethnicity or microbiome [37]. Further, and likely most importantly, their participants provided follow-up exposure information every second year in comparison to our study where exposure only was assessed at baseline. A number of prospective studies detected no association between saturated fatty acids and Parkinson disease [3, 17–19, 21, 23], nor did one case control study [22] and two meta-analyses [24, 38].

We further did not find any association between total fat, poly- or monounsaturated fat intake and Parkinson disease in our study. This is in line with findings from a prospective study by Chen et al. [21], as well as an additional prospective study [3] and one case control study on monounsaturated fat intake [22]. A few studies have shown a protective association between Parkinson disease and total fat intake [39], polyunsaturated fat intake [3, 16, 18, 23] and monounsaturated fat intake [17, 23] in contrast to our findings. However, the most recent meta-analysis [24] comprising seven studies showed a too weak association for both total and any subgroups of fat to draw a robust conclusion about the risk of Parkinson disease. The heterogeneity found in these epidemiologic studies may be explained by differences in study populations, e.g. different lifestyle and dietary habits due to origin (e.g. western vs. eastern diet). Moreover, difference in study design ranging from exposure assessment from different questionnaires, to how diagnosis of Parkinson disease was ascertained varied between studies. The response to dietary fat or the risk of Parkinson disease itself may also be modified by exposure to environmental neurotoxicants or genetic susceptibility.

The molecular mechanisms possibly linking Parkinson disease and saturated fatty acids are largely unknown. A high energy intake has been suggested to be associated with development of Parkinson disease [24]. However, when we additionally adjusted our model for total energy intake, we did not find a significant effect. Also, no association was found between total fat intake and Parkinson disease in the present study. Taken together, the absence of an association between total energy and total fat intake in relation to Parkinson disease incidence emphasizes the importance of saturated fat, specifically in Parkinson disease pathogenesis.

Saturated fatty acids are found in dairy products i.e. butter, cream and cheese, and in red meat. Saturated fat intake likely contribute to chronic inflammation and oxidative stress [11]. It is rather unlikely that this effect occurs from self-synthesized fatty acids and not from dietary fat intake. Saturated fat also affects the unsaturated fatty acids metabolism by creating adverse changes in the lipid composition of the cell membrane, including those in the brain [5, 36]. The lipid composition of the neuronal cell membranes plays a role in the susceptibility of oxidative stress [36].

Degeneration of dopamine neurons in striatal structures of the brain such as the substantia nigra is the hallmark of Parkinson disease. This degeneration is mediated by pro-inflammatory cytokines [10, 11, 40–42] yet the cause of the degeneration is unknown. Reduction of the enzyme tyrosine hydroxylase, which is rate-limiting in the dopamine synthesis, and dramatic loss of dopaminergic neuronal function has been shown in mice given a high-fat diet [43]. Lipid droplets storing fatty acids seem to be linked to obesity-driven metabolic dysregulation [44]. In the brain lipid droplet lipolysis has been suggested to depend on proper function of α-synuclein, a protein found aggregated in the brain in Parkinson disease [45]. The lipid-binding properties of α-synuclein are not fully understood, however the α-synuclein neurotoxicity may be independent of the aggregation process and more related to lipid membrane interactions, a process promoted by certain lipids and fatty acids, to the extent that some authors describe Parkinson disease as a lipidopathy [45]. Exposure to neurotoxic agents such as pesticides, including paraquat and rotenone, has also been suspected to contribute to Parkinson disease [46].

Pesticide exposure by individuals with a low intake of polyunsaturated fat, or a high intake of saturated fat in relation to unsaturated fat, seems to induce a specific vulnerability of striatal neurons and affect Parkinson disease risk [16]. Many pesticides are organic molecules with a metal species in the structure [47] where metals are known to enhance oxidative stress [48]. Oxidative damage to dopamine producing cells in the striatum may also be induced by metals [46, 49] including iron necessary for proper activity of tyrosine hydroxylase [46]. In conclusion, saturated fat exacerbating the effect of neurotoxicants, combined with a vulnerability to neurotoxicants due to neuroinflammation, may contribute to the observed association between saturated fat and Parkinson disease.

The present study has several strengths. First, its large sample size of 41,597 women and men, who were followed-up with regards to Parkinson disease in national registries for almost 20 years, which is of importance to detect cases of Parkinson disease as the incidence of Parkinson disease is quite low. Second, its prospective design, which allowed to accurately assess participants’ dietary habits before diagnosis of Parkinson disease. This limited the risks of selection or recall bias, the latter especially important in case of long prodromal phase of Parkinson disease. Moreover, we were able to determine dietary habits at baseline using the validated FFQ [27]. Third, we extensively adjusted for confounders. Finally, although the study cohort might be affected by a potential healthy volunteer bias, which could reduce generalizability of our findings, we believe our study has good external validity, given the large cohort with an essentially complete follow-up and the inclusion of participants of various demographic backgrounds believed to have provided valid information at baseline, with a low proportion of missing or incomplete data [25].

The limitations of the present study are the following: first, dietary intake was assessed only once at baseline through our semi-quantitative questionnaires with frequency category questions. This entails that (1) the true intake may not correspond to the one reported by the participant and might also be limited by incomplete food lists, a lack of information on food sources, cooking methods and use of standardized portion sizes, and (2) changes in dietary habits during the follow-up period could not be assessed. A possible consequence thereof, given our prospective study design, is non-differential misclassification of nutrient intakes. This would, if it occurred, most likely lead to an underestimation of the true association between nutrient intakes and the risk of Parkinson disease. Further, information on participants’ dietary habits was self-reported over long periods (i.e. weeks, months, years).

By applying the nutrient residual method and properly considering total energy intake in the analyses, we controlled for confounding and largely removed the extraneous variation, as well as reduced measurement error [29]. Nevertheless, residual confounding by unknown risk factors cannot be ruled out.

No clinical information on the phenotype of Parkinson disease at disease onset is available in the Patient Register. Moreover, it should be noted that although the national Inpatient Register has been shown to be a valid source to identify Parkinson disease cases in epidemiologic etiology studies, misclassification between Parkinson disease and other parkinsonian disorders were likely common [26]. Parkinsonism may be a sign of prodromal Parkinson disease, leading to an early diagnosis. At the same time, using ICD codes for diagnosis may have delayed diagnosis date. Data collection of outpatient hospital care did not start until 2001 [26], thus we lack outpatient information for the first four years of follow-up. In addition, prodromal symptoms preceding Parkinson disease at baseline might have caused changes in dietary habits, increasing the risk of reverse causality. It has been shown that the lack in dopamine has an impact on the eating behavior of patients affected by Parkinson disease [50]. Nevertheless, our findings were not essentially affected when we investigated potential effects of reverse causation in sensitivity analyses after removing cases occurring during the first three years of follow-up. The slightly attenuated effect after removing cases during the first five years of follow-up might be explained by reduced power as only 331 cases remained in the multivariable analyses after adjusting for confounders.

In conclusion, our results suggest that a higher intake of saturated fat might be associated with an increased risk of Parkinson disease. No association was found with total, monounsaturated or polyunsaturated fat intakes or by replacing total fat intake or specific types of fats by other macronutrients or fats, respectively. Dietary choices might have an influence on the development of Parkinson disease and limiting the intake of saturated fat may be beneficial for disease prevention.

## Supplementary Information

Below is the link to the electronic supplementary material.

Supplementary file1 (DOCX 10789 kb)

## Abbreviations

BMI: Body Mass Index
FFQ: Food Frequency Questionnaire
HR: Hazard Ratio

## Declarations

### Data Availability

Requests for anonymized data not published within this article should be addressed to the principal investigators of the Swedish National March Cohort (YTL, WY, and RB) and data will be made available by request from any qualified investigator.
