# Supplementary material for: Dietary fat intake and risk of Parkinson disease: results from the Swedish National March Cohort
Source: Eur J Epidemiol. 2022 Apr 13;37(6):603–13. doi: 10.1007/s10654-022-00863-8 (PMC9288363; doi:10.1007/s10654-022-00863-8)
Supplement: Supplementary file 1 — Supplementary file1 (DOCX 10789 kb) [file 10654_2022_863_MOESM1_ESM.docx]

**
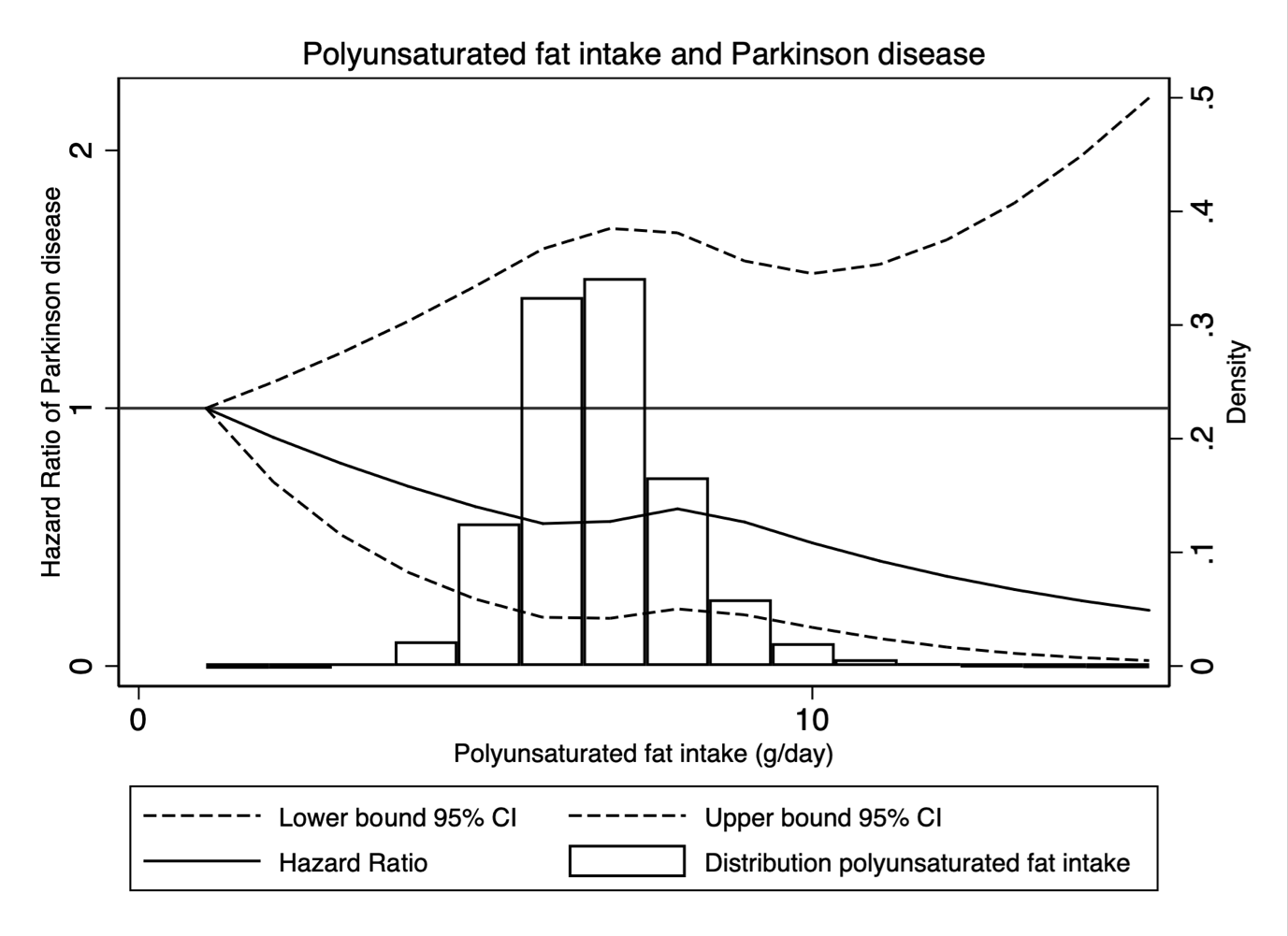
**

**Figure S1. Multivariable-adjusted restricted cubic spline curve for the association between dietary intake from polyunsaturated fat, measured in g/day, and the risk of Parkinson disease. Adjustments were made for age (underlying time scale), sex, intake of coffee (categories of 0, 1-2, 3-4, and ≥5 cups/day), dietary intake of Vitamin E (mg/day), Body mass index (BMI, kg/m2), education (≤13 or >13 years), smoking status (never, former or current) and physical activity, including household and commuting activity (categories of ≤2, 3-4, 5-6, >6 hours/week).**

Please add: and total energy intake (kcal/day)

**
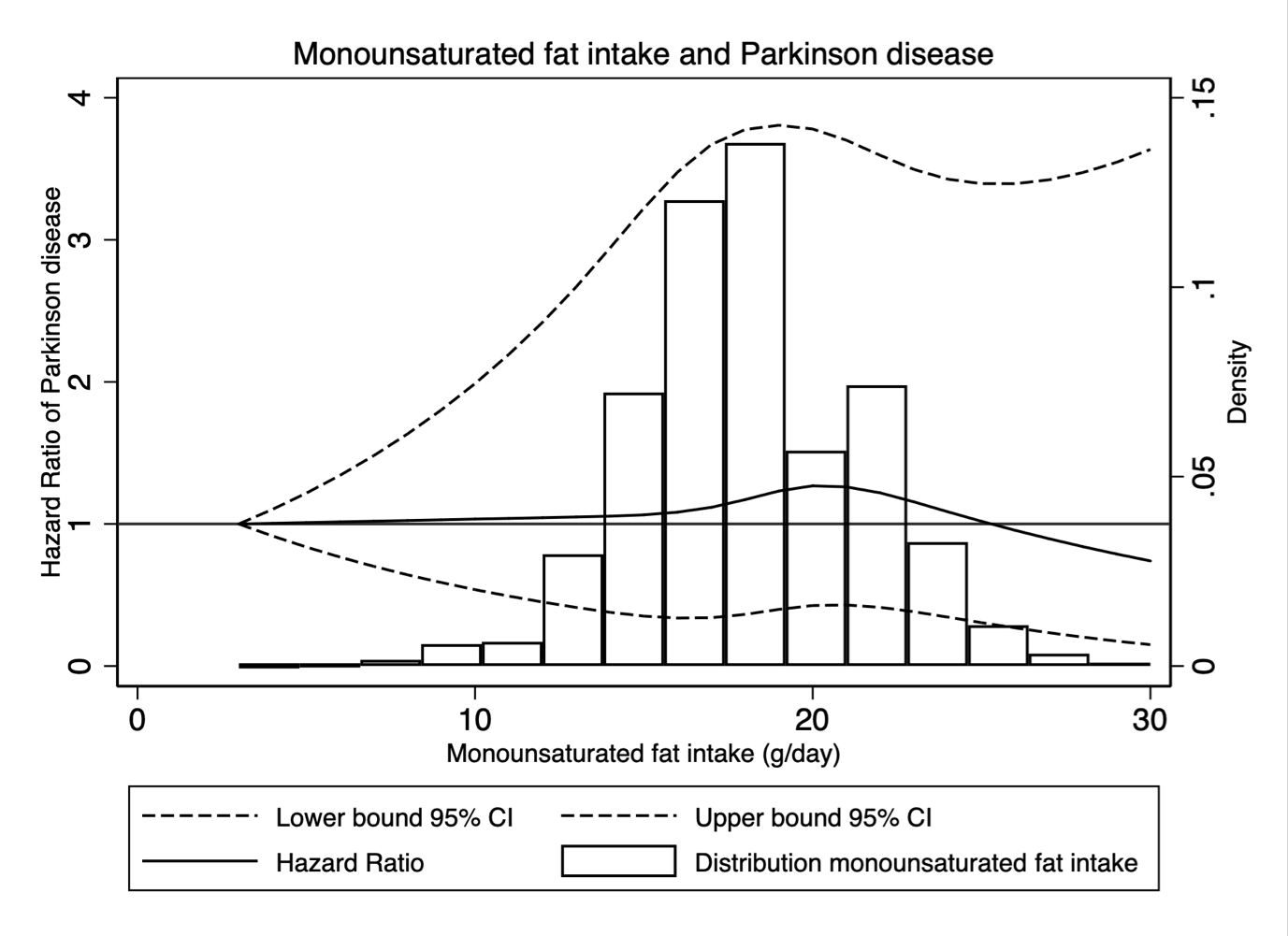
**

**Figure S2. Multivariable-adjusted restricted cubic spline curve for the association between dietary intake from monounsaturated fat, measured in g/day, and the risk of Parkinson disease. Adjustments were made for age (underlying time scale), sex, intake of coffee (categories of 0, 1-2, 3-4, and ≥5 cups/day), dietary intake of Vitamin E (mg/day), Body mass index (BMI, kg/m2), education (≤13 or >13 years), smoking status (never, former or current) and physical activity, including household and commuting activity (categories of ≤2, 3-4, 5-6, >6 hours/week).**

Please add: and total energy intake (kcal/day)
